# Human Cytomegalovirus miR-UL112-3p Targets TLR2 and Modulates the TLR2/IRAK1/NFκB Signaling Pathway

**DOI:** 10.1371/journal.ppat.1004881

**Published:** 2015-05-08

**Authors:** Igor Landais, Chantel Pelton, Daniel Streblow, Victor DeFilippis, Shannon McWeeney, Jay A. Nelson

**Affiliations:** 1 Vaccine and Gene Therapy Institute, Oregon Health and Science University, Portland, Oregon, United States of America; 2 Division of Biostatistics, Public Health and Preventive Medicine, Oregon Health and Science University, Portland, Oregon, United States of America; University of North Carolina at Chapel Hill, UNITED STATES

## Abstract

Human Cytomegalovirus (HCMV) encodes multiple microRNAs (miRNAs) whose functions are just beginning to be uncovered. Using *in silico* approaches, we identified the Toll-Like Receptor (TLR) innate immunity pathway as a possible target of HCMV miRNAs. Luciferase reporter assay screens further identified TLR2 as a target of HCMV miR-UL112-3p. TLR2 plays a major role in innate immune response by detecting both bacterial and viral ligands, including HCMV envelope proteins gB and gH. TLR2 activates a variety of signal transduction routes including the NFκB pathway. Furthermore, TLR2 plays an important role in controlling CMV infection both in humans and in mice. Immunoblot analysis of cells transfected with a miR-UL112-3p mimic revealed that endogenous TLR2 is down-regulated by miR-UL112-3p with similar efficiency as a TLR2-targeting siRNA (siTLR2). We next found that TLR2 protein level decreases at late times during HCMV infection and correlates with miR-UL112-3p accumulation in fibroblasts and monocytic THP1 cells. Confirming direct miR-UL112-3p targeting, down-regulation of endogenous TLR2 was not observed in cells infected with HCMV mutants deficient in miR-UL112-3p expression, but transfection of miR-UL112-3p in these cells restored TLR2 down-regulation. Using a NFκB reporter cell line, we found that miR-UL112-3p transfection significantly inhibited NFκB-dependent luciferase activity with similar efficiency as siTLR2. Consistent with this observation, miR-UL112-3p transfection significantly reduced the expression of multiple cytokines (IL-1β, IL-6 and IL-8) upon stimulation with a TLR2 agonist. Finally, miR-UL112-3p transfection significantly inhibited the TLR2-induced post-translational activation of IRAK1, a kinase located in the upstream section of the TLR2/NFκB signaling axis. To our knowledge, this is the first identified mechanism of TLR2 modulation by HCMV and is the first report of functional targeting of TLR2 by a viral miRNA. These results provide a novel mechanism through which a HCMV miRNA regulates the innate immune response by down-regulating TLR-2 expression.

## Author Summary

Human cytomegalovirus (HCMV) is a herpesvirus that is a leading cause of congenital defects in newborns and can be deadly in people with weakened immunity. HCMV has developed multiple strategies to escape the host immune system. Among those, microRNAs (miRNAs) are short regulatory RNAs that target gene transcripts through sequence complementarity. HCMV expresses more than 20 miRNAs and several of them, in particular miR-UL112-3p, have been demonstrated to cooperate in evading the host antiviral immune response during infection. In this work we identified TLR2, a cell surface receptor that plays an important role in the detection and control of CMV infection, as a novel target of miR-UL112-3p. We demonstrate that miR-UL112-3p efficiently down-regulates endogenous TLR2 during infection, causing significant inhibition of the downstream signaling cascade. This work provides the first identified mechanism of TLR2 modulation by HCMV and is the first report of TLR2 targeting by a viral miRNA.

## Introduction

The innate immune system is activated when microbial components (pathogen-associated molecular patterns or PAMPs) bind pattern recognition receptors (PRRs) located to the cell surface or in the intracellular compartment, leading to cellular changes including production of proinflammatory cytokines, increased motility and enhanced antigen presentation capabilities [1]. TOLL-like receptors (TLRs) are PRRs that play a critical role in controlling microbial infections. Each of the 10 TLRs identified in humans recognizes specific PAMPs, e.g. TLR4 binds Gram-negative bacteria lipopolysaccharides (LPS), TLR7/8 detects RNA virus infection by binding single-stranded RNAs, and TLR2 is responsive to bacterial lipoproteins through dimeric association with either TLR1 or TLR6 [2]. In the "classic" TLR2 pathway, binding of a PAMP to the receptor induces the recruitment of the adaptor protein MyD88 and IL-1 receptor-associated kinases (IRAK-4 and -1) via death domain interactions. The resulting phosphorylation and ubiquitination cascades activate the NFκB and MAP kinase (MAPK) pathways that in turn stimulate the transcription of various pro-inflammatory cytokines such as TNF-α, IL-6 and IFN-β [3].

In addition to bacterial lipopeptides, TLR2 is an important sensor of viral proteins including EBV dUTPase [4], Hepatitis C core and NS3 proteins [5] and Human Cytomegalovirus (HCMV) envelope glycoproteins B and H (gB and gH) [6,7]. HCMV gB and gH interact directly with TLR2 on the plasma membrane, resulting in the stimulation of the NFκB pathway in a MyD88-dependent manner and the production of inflammatory cytokines characteristic of innate immune detection. Interestingly, endosomal TLR2 was also shown to mediate expression of type I interferon in inflammatory monocytes upon murine CMV (MCMV) infection in a MyD88- and IRF3/IRF7 dependent manner [8].

Correlating with these *in vitro* studies, the biological importance of TLR2 to control CMV infection has been demonstrated *in vivo* in both human and mice. Single nucleotide polymorphism (SNP) analysis of human liver transplant recipients identified a frequent human TLR2 mutation that is a significant risk factor for HCMV reactivation and disease [9]. Further studies revealed that this mutation results in a functional defect of TLR2 stimulation and downstream signaling by HCMV [9–13]; in line with human data, MCMV infection of TLR2 knock-out (KO) mice led to elevated viremia in the spleen and liver compared with WT mice [14]. Taken together, these findings indicate that TLR2 plays a crucial role in the detection and control of CMV infection *in vivo*. However, despite its importance in the biology of CMVs, a viral mechanism for TLR2 modulation has not been reported.

MicroRNAs (miRNAs) are small non-coding RNAs that down-regulate target genes post-transcriptionally via incorporation into the RNA-induced silencing complex (RISC) (for review, see [15]). In addition to plants and animals, miRNAs have been identified in double-stranded, nuclear-replicating DNA viruses, and in particular herpesviruses (www.mirbase.org). A number of studies, including our own, have identified 24 mature miRNAs encoded by HCMV from 13 pre-miRNAs [16–18]. Unlike the miRNAs encoded by the α- and γ-herpesviruses, which form clusters in latency-associated regions of the genome, the HCMV-encoded miRNAs are located throughout the viral genome as both single miRNAs and small clusters [19,20]. Although miRNA sequences are not conserved between herpesviruses, the consensus in the field is that miRNAs from different viral species target similar genes or pathways in their respective hosts [21,22]. Herpesvirus miRNAs have been shown to target various aspects of virus and cell biology, including viral transcriptional activators and immune evasion genes [23–25] as well as cellular genes involved in cell cycle regulation, apoptosis, signal transduction and vesicular trafficking [26–32].

One of the most studied aspect of the biology of herpesvirus miRNAs is their role in the regulation of the host innate immune response. Very recently, our lab demonstrated that HCMV miR-US5-1, miR-US5-2 and miR-UL112-3p modulate inflammatory cytokine secretion by targeting multiple members of the secretome pathway, and that combined inactivation of these miRNAs in the virus disrupts the virus assembly complex (VAC) and causes severe growth defect [32]. Another HCMV miRNA, miR-UL148 was found to target the immune cell attracting chemokine RANTES [33]. Supporting the hypothesis that different viruses utilize distinct miRNA sets to modulate the same biologically important genes and pathways, work from the Mandelboim lab revealed that HCMV miR-UL112-3p as well as KSHV and EBV miRNAs all directly target MICB, a stress-induced ligand of the natural killer (NK) cell activating receptor NKG2D that plays an important role in the NK-mediated antiviral response [21,34,35]. In another example of functional convergence, various viruses have recently been found to modulate the TLR/ IL-1β pathway via miRNA targeting. Specifically, MyD88 and IRAK1 are directly targeted by KSHV miRNAs but also by a cellular miRNA, miR-21, that is induced during HCV, HIV-1 and VSV infection, resulting in reduced inflammatory cytokine and interferon expression [36,37].

Despite the fact that TLR2 is an important viral sensor that is regulated by cellular miRNAs [38–42], a viral miRNA has not been shown to target this gene. In this study we demonstrate that in addition to MICB and the secretome pathway, HCMV miR-UL112-3p modulates the TLR/IRAK1/NFκB signaling pathway by targeting TLR2, a HCMV sensor that plays an important role in mammalian control of CMV infection.

## Results

### TLR2 is targeted by HCMV miR-UL112-3p

To identify cellular pathways that are potentially targeted by HCMV miRNAs, we analyzed the reactome database (www.reactome.org, [43]) for pathways enriched in genes for which the 3'UTR contains one or more canonical HCMV miRNA target sites. Strikingly, several reactome pathways related to TLR signaling appeared to be targeted by HCMV miR-UL112-3p (S1 Fig, upper panel). Given the importance of the host innate immune response in the biology of herpesviruses and the fact that others have shown that TLR pathways are targeted by miRNAs during viral infection, we decided to explore this finding further.

**Fig 1:**
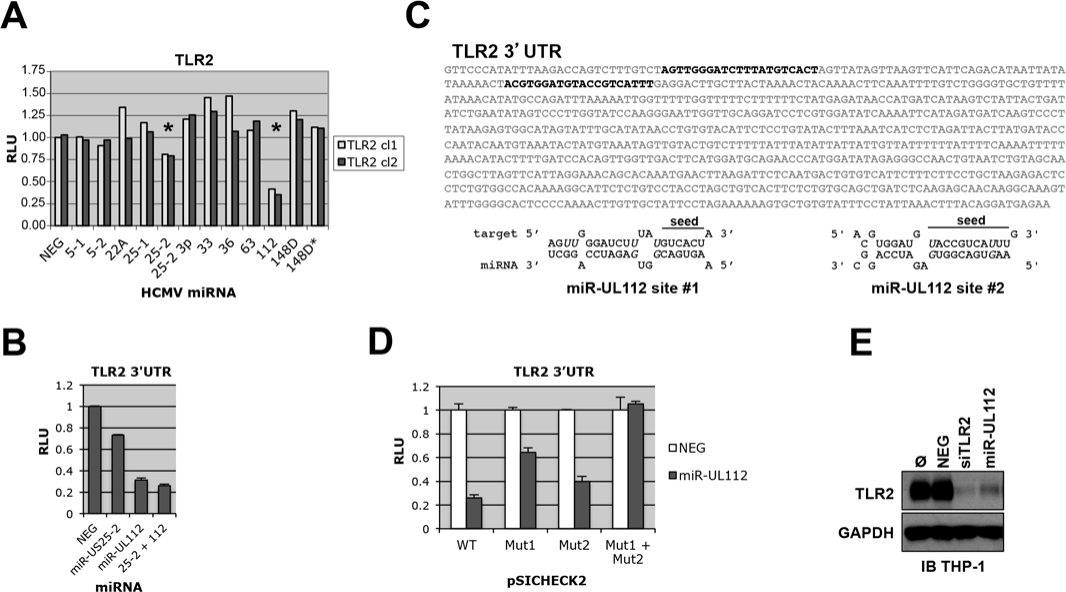
TLR2 3’UTR is targeted by HCMV miRNAs. (A) Dual luciferase reporter assay suggests that TLR2 3’UTR is targeted by miR-UL112-3p and miR-US25-2. Two independent pSICHECK2-TLR2 3’UTR clones were tested; stars indicate potential Renilla luciferase down-regulation. Putative and confirmed HCMV miRNAs are indicated in abbreviated form (see detailed list in legend of S1 Fig) (B) TLR2 3’UTR is down-regulated by miR-UL112-3p. HEK293 cells were transfected with pSICHECK2-TLR2 3’UTR and the indicated miRNA mimics alone or in combination. (C) Two potential miR-UL112-3p target site are present near the 5’end of TLR2 3’UTR (bold characters). Wobble G-U pairs are in italics; seed sequences are indicated. (D) Both target sites are required for full miR-UL112-3p down-regulation of TLR2. pSICHECK2 vectors containing mutations in target sites #1 and #2 (mut1 and mut2, respectively) were assayed by luciferase assay with the indicated miRNAs. (E) A miR-UL112-3p mimic down-regulates endogenous TLR2 in THP1 cells. TPA-differentiated THP1 cells left untransfected (Ø) or transfected as indicated were harvested 2 days post-transfection for IB analysis.

To determine experimentally if genes belonging to the TLR pathway are targeted by HCMV miRNAs, we cloned the 3'UTR of 10 human genes belonging to the pathway (TLR1, TLR2, TLR3, TLR9, CD14, Rac1, Rac2, Rac3, TICAM1, TICAM2) in the pSICHECK2 dual luciferase reporter construct downstream of the Renilla luciferase gene. Individual co-transfection of these constructs with 12 putative and confirmed HCMV miRNA mimics or a negative control in HEK293Tcells followed by luciferase assay suggested that TLR2 is targeted by two miRNAs, miR-US25-2 and miR-UL112-3p (Figs 1A and S1). miR-UL25-2 inhibited luciferase activity by approximately 20% while miR-UL112-3p had a stronger effect with 65% inhibition. None of the other TLR pathway genes were reproducibly down-regulated by the HCMV miRNA mimics tested in this assay (S1 and S2 Figs).

Since TLR2 3'UTR appears targeted by two miRNAs, we tested whether they could synergize in the down-regulation of the reporter gene. As shown in Fig 1B, miR-US25-2 did not exhibit significant effect in down-regulating the TLR2 3'UTR reporter when co-transfected with miR-UL112-3p. These results indicate that miRUL112-3p is solely responsible for TLR2 down-regulation. Taken together with the fact that miR-US25-2 transfection was unable to down-regulate TLR2 by immunoblot (IB) (S3 Fig), we chose to focus exclusively on miR-UL112-3p in subsequent experiments. Using RNAhybrid (http://bibiserv.techfak.uni-bielefeld.de/rnahybrid/) we identified two potential target sites for miR-UL112-3p located in the proximal region of TLR2 3’UTR and separated by 41bp (Fig 1C). Site-directed mutagenesis was performed on the seed sequence of each target site in the pSICHECK2-TLR2 3’UTR reporter construct, individually or in combination. Luciferase assay analysis indicated that mutation of individual target sites partially rescued the knockdown induced by miR-UL112-3p in the WT construct (40% rescue for site #1 and 15% rescue for site #2, Fig 1D), while the double mutation resulted in complete rescue (75% rescue compared to the WT construct). If the two sites had additive effect, the rescue would be approx. 40+15: 55%, which is significantly lower than the observed rescue by the double mutant. These results suggest that the down-regulation of TLR2 by miR-UL112-3p is mediated by two closely spaced target sites acting cooperatively. We next tested if transfection of a miR-UL112-3p mimic could target endogenous TLR2. For this experiment we used TPA-differentiated THP-1 cells in which TLR2 is easily detectable by IB (Fig 1E). Compared to untransfected cells or cells transfected with a non-targeting control siRNA (NEG), TLR2 expression was strongly reduced upon transfection of a miR-UL112-3p mimic. Strikingly, this mimic was almost as potent as a commercial siRNA against TLR2, suggesting that miR-UL112-3p targets TLR2 with siRNA-like efficiency (Fig 1E). This unusually high efficiency (for a miRNA) is probably due to the cooperative effect of the two target sites identified above.

These results indicate that miR-UL112-3p efficiently targets endogenous TLR2 via two target sites located in the proximal part of the 3'UTR sequence.

### TLR2 is down-regulated during HCMV infection

**Fig 2:**
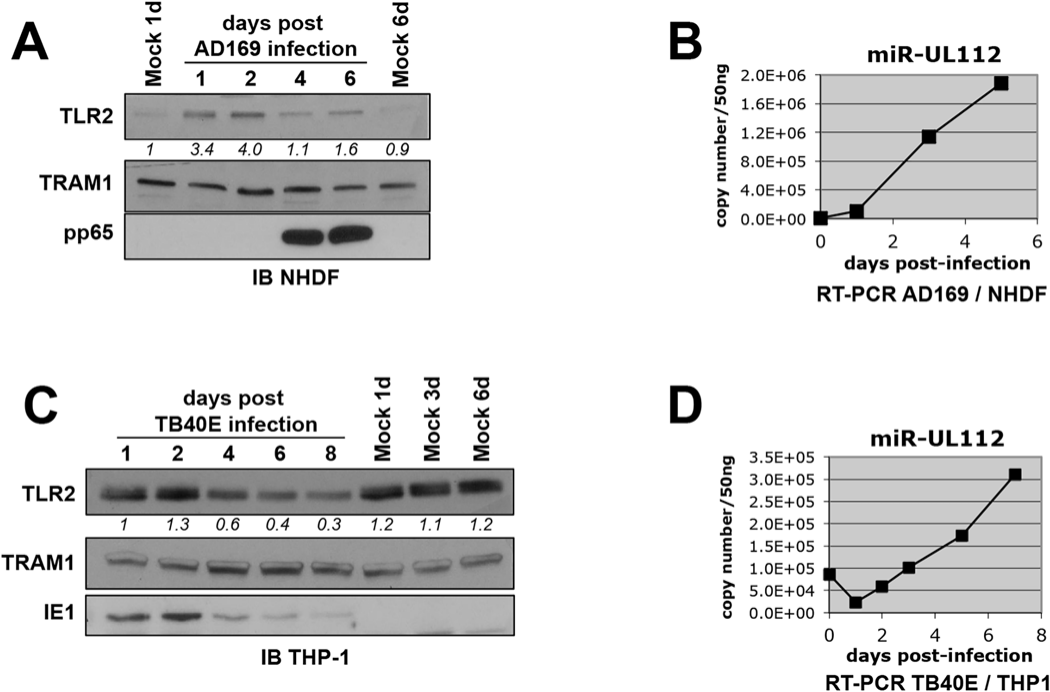
TLR2 is down-regulated during HCMV infection. (A) TLR2 follows a biphasic expression pattern during HCMV AD169 infection of NHDF fibroblasts with early induction and late down-regulation. Numbers below the TLR2 blot represent quantification of the protein signal. IB, immunoblot. TRAM1 was used as loading control. (B) miR-UL112-3p accumulates at late time points during AD169 infection of NHDF cells. miR-UL112-3p copy number was determined by RT-PCR at various points during the infection of NHDF fibroblasts with HCMV AD169 (MOI: 3). (C) TLR2 is down-regulated late during TB40E infection of THP-1 monocytic cells. (D) miR-UL112-3p accumulates at late time points during TB40E infection of differentiated THP-1 cells (MOI: 3).

Like most HCMV miRNAs, miR-UL112-3p is expressed with early kinetics during *in vitro* HCMV infection and accumulates over time [17]. We reasoned that miR-UL112-3p might down-regulate TLR2 as it accumulates during infection. To test this hypothesis, we first monitored the protein level of endogenous TLR2 during HCMV infection in productively infected fibroblasts by western blot (IB) (Fig 2A). Quantitation of TLR2 revealed that the protein follows a biphasic expression in fibroblasts infected with the lab strain AD169. First, TLR2 was significantly up-regulated within 24–48 hours post-infection (hpi). TLR2 upregulation upon infection with HCMV and other viruses such as HSV1 and Influenza has been observed by others both at the mRNA and protein level [44–47] and requires live virus since UV-inactivated virus is unable to induce TLR2 (S4 Fig and [45]). Early TLR2 accumulation was followed by a down-regulation phase clearly seen at 4 and 6dpi, which corresponded to the late phase of infection as indicated by the detection of pp65, a late gene product. TLR2 biphasic expression was not a loading artifact since probing the same blot for TRAM1, a protein that remains constant during HCMV infection, indicated fairly consistent loading between the lanes. In line with our hypothesis that miR-UL112-3p targets TLR2 during infection, the TLR2 decrease correlated temporally with the progressive accumulation of miR-UL112-3p in infected NHDF cells (Fig 2B). We performed similar experiment using TPA-differentiated THP-1 cells (a monocytic cell line) infected with the HCMV clinical strain TB40E. In contrast with fibroblasts, TLR2 was abundantly expressed in uninfected THP-1 and was not significantly upregulated upon infection. However, similar to fibroblasts, TB40E infection resulted in the progressive down-regulation of TLR2 at 4-6dpi (Fig 2C) that temporally correlated with the progressive accumulation of miR-UL112-3p in TB40E-infected cells (Fig 2D).

Taken together, these results show that TLR2 is down-regulated late during HCMV infection at time when miR-UL112-3p accumulates significantly.

### miR-UL112-3p down-regulates endogenous TLR2 during HCMV infection

**Fig 3:**
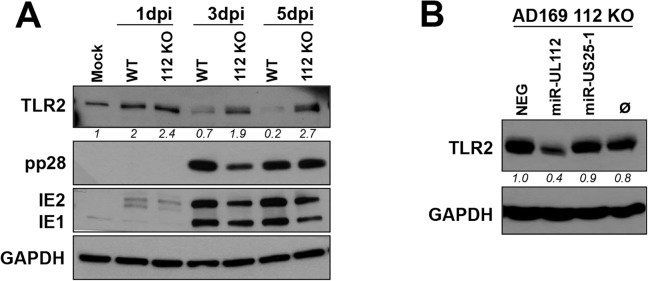
miR-UL112-3p targets endogenous TLR2 during HCMV infection. (A) A miR-UL112-3p deficient AD169 virus fails to down-regulate endogenous TLR2 during infection. NHDF cells infected (MOI: 1) with AD169 WT or a miR-UL112-3p mutant that does not express miR-UL112-3p were harvested at various times post-infection for IB analysis. (B) Transfection of a miR-UL112-3p mimic down-regulates TLR2 in NHDF cells infected with a miR-UL112-3p-deficient virus. NHDF cells were infected with AD169 112 KO (MOI: 3), left untransfected (Ø) or transfected 2 hrs later with various siRNA and miRNA mimics and harvested 2 dpi for IB analysis.

To determine whether miR-UL112-3p expressed by HCMV during infection is responsible for the down-regulation of TLR2 shown in Fig 2, we used an AD169 miR-UL112-3p KO mutant BAC construct [32]. This mutant contains seven silent mutations in the miR-UL112-3p locus that disrupt the secondary structure of the miRNA resulting in the loss of miR-UL112-3p expression without affecting expression of UL114 located on the complementary strand [32]. Side-by-side infection experiments using AD169 WT and AD169 miR-UL112-3p KO in fibroblasts revealed that both viruses induce TLR2 with similar efficiency at 1dpi (Fig 3A). Strikingly, in contrast with WT virus infection in which TLR2 level decreased over time, TLR2 levels remained stable 3 and 5 days post-infection with the miR-UL112-3p-deficient virus. Similar experiment performed with a triple miRNA mutant virus that does not express miR-UL112-3p, miR-US5-1 and miR-US5-2 [32] gave the same result (S5 Fig). This effect was not due to an infection defect by the mutant viruses since viral proteins IE1, IE2, pp28 and pp65 were expressed at levels comparable to the WT virus throughout infection (Figs 3A and S4 and S5). Furthermore, we and others have demonstrated that AD169 miR-UL112-3p KO does not display any significant growth defect compared to WT virus in fibroblasts [32,48]. These data demonstrate that miR-UL112-3p expression is required for TLR2 down-regulation during HCMV infection.

Since the level of TLR2 is maintained in cells infected with a miR-UL112-3p-deficient virus, we next tested whether transfection of exogenous miR-UL112-3p could reverse this effect (Fig 3B). NHDF cells were infected with AD169 miR-UL112-3p KO, transfected with siRNA/miRNA mimics 2 hrs later and harvested 2 dpi. Transfection of the miR-UL112-3p mimic markedly reduced the level of TLR2 compared to untransfected cells or cells transfected with miRNA mimics that do not target TLR2 (NEG and miR-US25-1).

Taken together, these results indicate that miR-UL112-3p expressed by HCMV during infection efficiently down-regulates endogenous TLR2.

### miR-UL112-3p inhibits TLR2 signaling and NFκB activation

**Fig 4:**
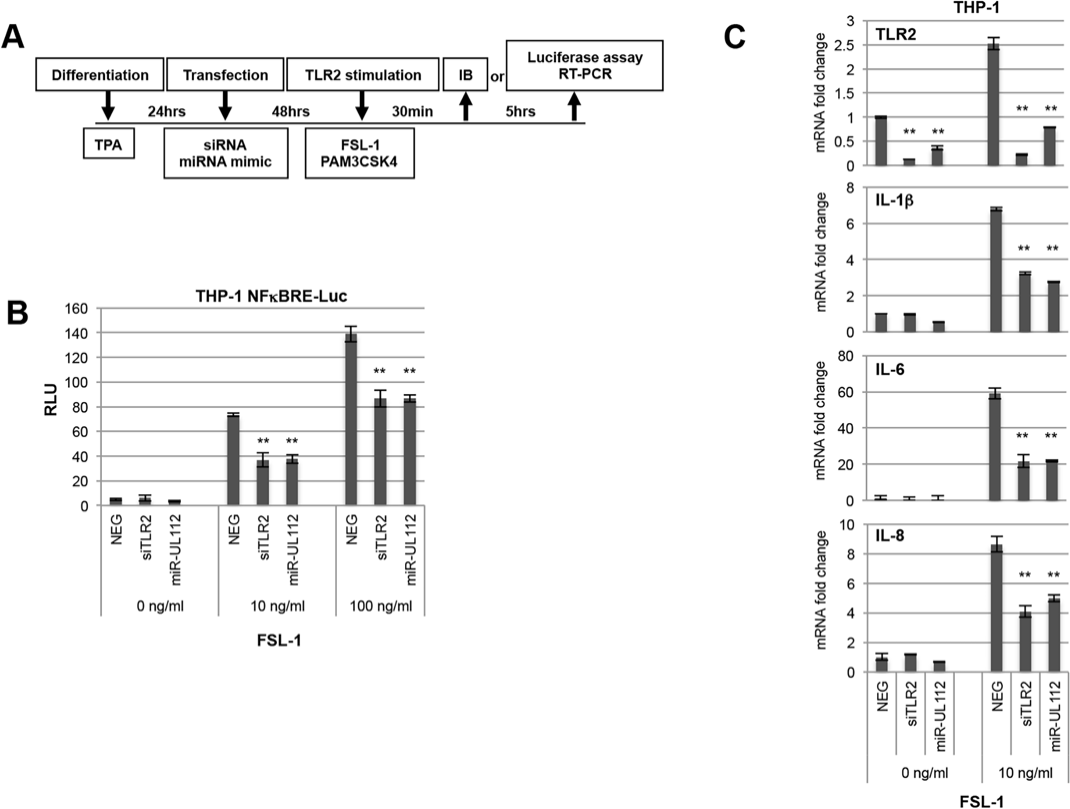
miR-UL112-3p inhibits TLR2-dependent activation of the NFκB pathway in THP1 cells. (A) Schematic of cell treatment. THP1 or NFκB luciferase reporter THP1 cells (THP1-NFκBRE-luc) were transfected with siRNA or miRNA mimics after being differentiated for 24hrs with TPA. Cells were stimulated 48hrs post-transfection with the TLR2/TLR6 agonist FSL-1 and harvested either 30 min or 5 hrs later for IB analysis or luciferase assay, respectively. (B) miR-UL112-3p inhibits NFκBRE-driven luciferase activity stimulated by the TLR2 agonist FSL-1 with similar efficacy as siTLR2. Each test was performed in quadruplicate. (C) miR-UL112-3p decreases TLR2 mRNA level (upper panel) and IL-1β, IL-6 and IL-8 mRNA expression stimulated by the TLR2 agonist FSL-1 (three lower panels) with similar efficacy as siTLR2. Each test was performed in duplicate. **, P-value <0.0001 (sample vs. NEG).

HCMV inhibits the NFκB pathway late in infection, presumably to repress the expression of cytokines and chemokines by the infected cell [49]. Since TLR2 is a major PRR trigger of NFκB signaling, we tested whether miR-UL112-3p inhibits TLR-2-dependent NFκB signaling. These experiments were performed in differentiated THP-1 cells using the outline described in Fig 4A. First, we evaluated the impact of miR-UL112-3p on the induction of NFκB expression in THP1 reporter cells expressing luciferase under the control of the NFκB Response Element (THP1-NFκBRE-luc) upon stimulation with the TLR2/TLR6 agonist FSL-1 (Fig 4B). FSL-1 efficiently induced luciferase expression in cells transfected with NEG (15-fold and 28-fold at 10 and 100ng/ml FSL-1 over non-stimulated cells, respectively). Knocking down TLR2 with a siRNA (siTLR2) inhibited luciferase activity by 50% at 10ng/ml FSL-1 and by 40% at 100ng/ml FSL-1. Transfection with miR-UL112-3p mimic had the same effect as the siRNA, suggesting that miR-UL112-3p efficiently inhibited TLR2-mediated NFκB activation.

We next monitored the effect of siTLR2 and miR-UL112-3p on the expression of endogenous cytokines in THP-1 cells using the same experimental outline (Fig 4A). In these experiments, mRNA expression of TLR2 and three NFκB-responsive cytokines that play a significant role in the HCMV life cycle (IL-1β, IL-6 and IL-8) was monitored by quantitative RT-PCR upon stimulation with FSL-1 (Fig 4C). First, we evaluated the impact of FSL-1, siTLR2 and miR-UL112-3p on TLR2 mRNA level (top panel). While TLR2 transcription was mildly induced (x 2.5) by FSL-1 in cells transfected with NEG, TLR2 mRNA level was strongly reduced in cells transfected with siTLR2 (90% reduction) and slightly less with miR-UL112 (70% reduction). These results correlated well with the effect of siTLR2 and miR-UL112-3p on TLR2 protein level in THP-1 cells (Figs 1E and S7), suggesting that miR-UL112-3p mainly targets TLR2 via a direct mRNA degradation mechanism rather than post-translational silencing.

The three lower panels of Fig 4C indicate that, compared to unstimulated cells, FSL-1 robustly induced IL-1β, IL-6 and IL-8 transcription in cells transfected with NEG. Similar to NFκBRE reporter cells, knocking down TLR2 with siTLR2 inhibited the induction of IL-1β, IL-6 and IL-8 by 50–60% compared to NEG transfection. Transfection with miR-UL112-3p had the same effect as the siRNA, confirming that miR-UL112-3p efficiently inhibits TLR2-mediated activation of endogenous NFκB responsive genes.

**Fig 5:**
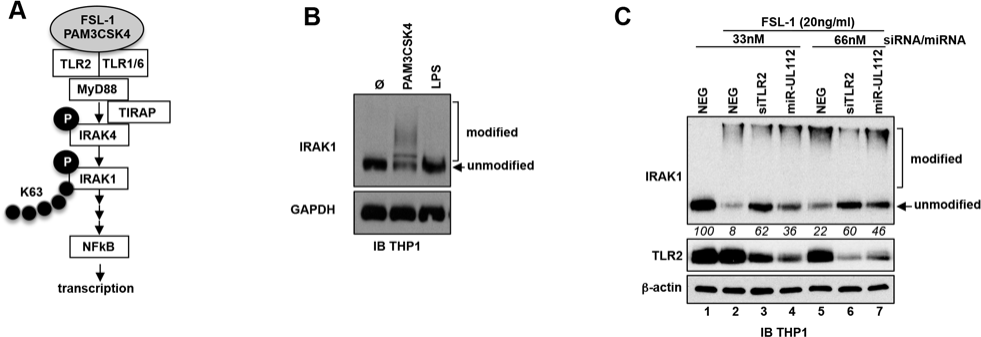
miR-UL112-3p inhibits TLR2-dependent activation of IRAK1. (A) Schematic of the upper section of the TLR2/NFκB pathway with known factors and post-translational modifications of IRAK4 and IRAK1 upon stimulation with TLR2 agonists. Grey ellipse, agonist; P, phosphorylation; K63, Lys63 poly-ubiquitin chain. (B) IRAK1 undergoes post-translational modifications upon stimulation with a TLR2/TLR1 agonist (PAM3CSK4, 100ng/ml) but not with a TLR4 agonist (LPS). TPA-differentiated THP-1 cells were treated as described in panel A. Unmodified and post-translationally modified forms of IRAK1 were detected by IB. (C) miR-UL112-3p inhibits FSL-1 induced IRAK1 post-translational modifications. TLR2, β-actin and IRAK1 unmodified and modified forms were detected by IB. Numbers below the IRAK1 blot represent quantification (in relative units) of the unmodified protein signal.

Albeit the effect of siTLR2 on NFκB signaling stimulated by a TLR2 agonist can be explained by the knock-down of TLR2, we cannot exclude a composite effect in the case of miR-UL112-3p since miR-UL112-3p might target other members of the NFκB pathway. We therefore tested whether miR-UL112-3p could inhibit TLR2 signaling upstream of the IKK convergence point of the NFκB pathway. As depicted in Fig 5A, upon agonist stimulation, TLR2 in association with TLR1 or TLR6 signals to IRAK4 via interaction with MyD88 (and in some conditions TIRAP/MAL). This interaction results in the phosphorylation and activation of IRAK4 that in turn phosphorylates IRAK1. In the process, IRAK1 is also polyubiquitinated with Lys-63 linkage, which does not lead to proteasomal degradation of the protein. These extensive IRAK1 post-translational modifications induce the rapid formation of higher molecular weight forms along with a marked reduction of the non-modified form that can be detected by IB in THP-1 cells treated with PAM3CSK4 (Fig 5B) and FSL-1 (Fig 5C). Comparison between these 2 agonists revealed that FSL-1 is more efficient than PAM3CSK4 in inducing IRAK1 modifications (Fig 5, compare panels B and C); FSL-1 was therefore used in subsequent experiments. In contrast with TLR2 agonists, treatment of THP-1 cells with the TLR4 agonist LPS failed to induce IRAK1 modifications (Fig 5B) due to the fact that uninfected THP1 cells express low levels of TLR4 [50]. Accordingly, LPS was much less efficient than PAM3CSK4 in activating NFκBRE in THP1-NFκBRE-luc cells, an effect opposite to fibroblast- NFκBRE-luc cells that do express TLR4 (S6 Fig, [51]). Taken together, these results indicate that detection of IRAK1 activation by IB is a good proxy to monitor the activation of the upstream part of the TLR2 pathway. Practically, we found that comparing the unmodified form of IRAK1 between treatments was a more robust indicator of IRAK1 activation than measuring the modified forms, which in our IB experiments did not correlate with the actual amount of IRAK1 post-translational modifications (Fig 5C). We next transfected THP-1 cells with NEG, siTLR2 or miR-UL112-3p at two different concentrations for two days then monitored the level of TLR2 and IRAK1 modifications by IB after stimulation with FSL-1 (Fig 5C). TLR2 was unaffected by FSL-1 treatment (lanes 1 and 2) but was efficiently down-regulated by siTLR2 and miR-UL112-3p (lanes 3–4 and 6–7). IRAK1 was efficiently modified upon FSL-1 treatment as indicated by the 92% and 78% decrease of the unmodified form (as measured in relative units, RU) in cells transfected with 33nM and 66nM NEG, respectively (compare lanes 1, 2 and 5). The unmodified form of IRAK1 was more abundant in cells transfected with siTLR2 than NEG at all siRNA/miRNA concentrations (62 vs. 8 and 60 vs. 22 RU, respectively), confirming that knocking down TLR2 reduced IRAK1 activation triggered by FSL-1. miR-UL112-3p also reduced IRAK1 activation but it did so less efficiently than siTLR2 at all siRNA/miRNA concentrations (36 vs. 62 and 46 vs. 60 RU), even though TLR2 knock-down by siTLR2 and miR-UL112-3p was similar (lanes 3–4 and 6–7). This effect was not due to direct targeting of IRAK1 by miR-UL112-3p as IRAK1 level was not affected by miR-UL112-3p transfection in the absence of TLR2 stimulation (S7 Fig). The fact that miR-UL112-3p did not inhibit IRAK1 activation as efficiently as siTLR2 but was nevertheless as efficient in inhibiting NFκB activation (Fig 4B and 4C) suggests that miR-UL112-3p targets additional NFκB signaling factors downstream of IRAK1.

Taken together, these results suggest that by targeting TLR2 and probably other members of the NFκB pathway, miR-UL112-3p inhibits NFκB pathway signaling triggered by TLR2 agonists.

## Discussion

In this paper we show that miR-UL112-3p efficiently targets TLR2 during HCMV infection, resulting in the inhibition of TLR2-mediated NFκB signaling. Several viruses have been shown to inhibit TLR2 signaling, including VZV [52,53], HSV [54] and HBV [55,56]. TLR2 itself is down-regulated at the transcriptional level during HSV2 infection [57], underlining the importance of this innate immune sensor to control viral infection. However, although TLR2 is targeted by several human miRNAs including miR-105, miR-19, miR-1225-5p, miR-143 and miR-154 in a variety of cell types [38–42], no viral miRNA has been shown to target TLR2—until now.

*In silico* analysis coupled with luciferase assays screens identified TLR2 as a potential target of HCMV miR-UL112-3p and miR-US25-2. Additional luciferase assays revealed that miR-UL112-3p was much more efficient than miR-US25-2 at targeting TLR2 3'UTR, with the latter having negligible effect in combination analysis. Confirming these findings, transfection of a miR-US25-2 mimic in fibroblasts had no effect on TLR2 protein level (S3 Fig). In contrast, transfection experiments in fibroblasts and THP-1 cells revealed that miR-UL112-3p alone down-regulates TLR2 with similar efficiency as a commercial TLR2 siRNA (Figs 1E and 4E and S3). Two functional, closely spaced miR-UL112-3p target sites were identified in the proximal region of TLR2 3'UTR and mutagenesis experiments demonstrated that both sites are required for full miR-UL112-3p targeting. Site #1, which individually provides the most efficient targeting, contains a canonical 6-mer seed sequence and extensive 3’ compensatory pairing; site #2, which is less efficient than site #1, contains an extensive non-canonical, 11-mer seed sequence that contains two G:U wobbles and additional 3’ compensatory pairing. Our mutagenesis data further suggests that the two miR-UL112-3p target sites have a cooperative effect, a phenomenon previously described for closely spaced miRNA target sites [58]. Importantly, synergy between the two target sites probably explains the siRNA-like efficiency of miR-UL112-3p in down-regulating TLR2 at the protein level.

To assess the functional effect of miR-UL112-3p down-regulation of TLR2, we tested whether NFκB pathway activation by TLR2 agonists is affected by miR-UL112-3p transfection (Figs 4 and 5). In addition to NFκB, TLR2 signals through other pathways including P38, JNK and Interferon Response Factors (IRFs) (for review, [3]) that could potentially play a major role in the TLR2-dependent modulation of HCMV infection. Nevertheless, in this study we focused on the NFκB pathway because of its demonstrated importance in CMV biology and the fact that it is a well-studied TLR2 signaling route with many robust in vitro functional assays available. NFκB reporter assays, endogenous cytokine expression and IRAK1 post-translational modifications allowed us to monitor activation of NFκB signaling at two different points in the pathway, one in the early/upstream phase of signaling (IRAK1) and one in the late/downstream phase that corresponds to the transcriptional activity of NFκB. Because miR-UL112-3p could in principle target multiple members of the NFκB pathway in addition to TLR2, being able to assess its impact at different steps of the signaling route was important to evaluate the contribution of TLR2 targeting to the overall effect. Using these assays, we also compared the efficiency of PAM3CSK4 with another TLR2 agonist, FSL-1. We found that FSL-1, a TLR2/TLR6 agonist, induced more efficiently and robustly IRAK1 modification and NFκBRE-mediated luciferase production than PAM3CSK4, a TLR2/TLR1 agonist (Fig 5, compare panels B and C), perhaps due to higher expression or better availability of TLR6 on the THP-1 cell surface. Based on this finding we used FSL-1 in subsequent experiments.

Using NFκBRE-Luc reporter assays, we found that miR-UL112-3p inhibited the FSL-1 dependent activation of NFκB and NFκB-dependent cytokines by 40–50%, similar to the effect observed with a TLR2 siRNA (Fig 4B and 4C). However, miR-UL112-3p was not as efficient as siTLR2 in preventing IRAK1 modification (Fig 5C), suggesting that miR-UL112-3p targets additional members of the NFκB pathway downstream of IRAK1. Could miR-UL112-3p also target additional genes upstream of IRAK1? Most known TLR2 pathway genes upstream of IRAK1 either do not contain a canonical miR-UL112-3p target site in their mRNA sequence (CD14, TLR1, TIRAP/MAL, IRAK4) or are not targeted by miR-UL112-3p in experimental setting (MyD88, S3 Fig). TLR6 mRNA contains two sites complementary to miR-UL112-3p seed sequence and remains a possible target. However, the fact that siTLR2 inhibited IRAK1 modifications more efficiently than miR-UL112-3p while down-regulating TLR2 with similar efficiency argues against this possibility. Taken together, these data suggest that miR-UL112-3p modulation of IRAK1 activation occurs mainly through TLR2 down-regulation.

To explore the significance of miR-UL112-3p for the regulation of the TLR2→IRAK1→NFκB axis in the context of infection, we also attempted numerous infection experiments with WT and miR-UL112-3p mutant viruses in different conditions, including various cell types, virus strains, multiplicities of infection (MOIs), timepoints and TLR2 signaling stimuli. These experiments revealed that, in an *in vitro* setting, cells become permanently unresponsive to any TLR2 stimulation in a matter of hours after HCMV infection. This unresponsiveness, which is independent of TLR2 levels (see Fig 2A and 2C), is probably mediated by early viral factors acting downstream of TLR2. Consequently, since significant targeting of TLR2 by miR-UL112-3p does not occur earlier than 2–3 days post-infection—long after the cells have become unresponsive to TLR2 signaling—we could not test how lack of miR-UL112-3p affects TLR2 signaling (including IRAK1 and NFκB activation) in the context of HCMV infection. In the absence of such experiments, we cannot formally rule out that miRNA-mediated manipulation of TLR2 has non-NFκB effects, or even no consequential effect, relevant to HCMV infection. Stemming from this uncertainty, other miR-UL112-3p targets such as MICB [21], members of the secretory pathway [32] or yet unidentified genes may be more important “drivers” of miR-UL112 biology. However, recent work showing that TLR2 plays an important role in the control of CMV infection argues against this possibility. As mentioned in the introduction, binding of HCMV envelope glycoproteins gB and gH to TLR2 on the cell surface triggers the cellular innate response, resulting in activation of the NFκB pathway and the production of inflammatory cytokines [6,7,59]. This mechanism is similar in other herpseviruses, for instance HSV that stimulates TLR2 and the NFκB pathway via gH/gL [60]. The functional consequences of impaired TLR2 signaling on CMV infection have been established *in vivo* in both humans and mice: in humans, the most frequent single nucleotide polymorphism (SNP) occurring in TLR2, R753Q [11], is a risk factor for HCMV disease in liver transplant recipients [9,12]; The R753Q mutation was shown to impair tyrosine phosphorylation, dimerization with TLR6, and recruitment of Mal and MyD88, resulting in a functional defect of TLR2 stimulation [10,13]. Directly linking this SNP to CMV disease, Brown *et al*. [59] found that the R753Q mutation impairs gB-dependent TLR2 stimulation. In a TLR2 KO mouse model, MCMV infection led to elevated viremia in spleen and liver compared to WT mice [14]. This effect was associated with lower levels of IL18 and IFN-α/β along with a decreased NK cell population. Depletion of NK cells abolished the difference in viral titers between TLR2 KO and WT mice, suggesting that this effect was NK cell-dependent. These data could suggest that TLR2 signaling is important for NK cell-dependent control of MCMV infection. Interestingly, miR-UL112-3p has previously been shown to target the immune ligand MICB to escape recognition of HCMV-infected cells by NK cells [21,35]. Given that miR-UL112-3p directly targets TLR2 as shown here, we speculate that by targeting both TLR2 and MICB, miR-UL112-3p has evolved as a multi-pronged weapon for CMV to escape NK cell control.

In parallel with its well-documented stimulation of the NFκB pathway, which has pro-survival effects, TLR2 signaling has also been shown in some situations to trigger apotosis either via direct recruitment of FADD through MyD88 and activation of caspase 8 [61], or autocrine stimulation of TNF-α (for review, [62]). In the context of a HSV mouse infection model, Aravalli *et al*. [63] demonstrated that HSV infection induces microglia cell apoptosis in a TLR2-dependent manner. HCMV infection has also been found to trigger apoptosis in a TLR2-dependent manner: during congenital HCMV infection, placentae obtained from newborns often display chronic villitis and disruptions of the syncytiotrophoblast (ST) [64]. Binding of HCMV to TLR2 on the surface of ST cells increased secretion of TNF-α, causing apoptosis of non-infected neighboring ST cells [45,64,65]. To prevent apoptosis in infected cells, HCMV has developed several strategies including stimulation of pro-survival pathways such as the NFκB pathway early during infection along with expression of anti-apoptotic factors such as UL36 and UL37 [66,67]. As NFκB signaling is markedly down-regulated in the late phases of HCMV lytic infection [49], it is conceivable that the late down-regulation of TLR2 by miR-UL112-3p might contribute to keep the survival/apoptosis balance toward survival [61].

As discussed above, a potential proviral effect of TLR2 down-regulation by HCMV miR-UL112-3p is the prevention of cellular innate signaling stimulation triggered by gB and gH binding to TLR2 on the cell surface before viral entry. A major caveat to this hypothesis is that miR-UL112-3p is expressed after viral entry and TLR2 down-regulation occurs at late times of lytic infection. It is therefore unlikely that the virus inhibits the immediate innate response by this means. However, two possibilities could solve this apparent conundrum: first, we have detected the presence of miR-UL112-3p in highly purified infectious and non-infectious viral particles, opening the possibility that miR-UL112-3p could be delivered in the cell at the time of virus entry before productive infection even begins. Alternatively, evidence is mounting that cellular and viral miRNAs produced by uninfected or infected cells are actively secreted and stabilized by extracellular miRNA carriers (such as exosomes, apoptotic bodies and microvesicles) and can have a paracrine effect on neighboring or more distant cells. This new form of intercellular communication has been identified in multiple cell types and tissues (for review, [68,69]). For instance, exosomal miRNAs from the placenta-specific chromosome 19 miRNA cluster are secreted by trophoblasts and attenuate replication of multiple viruses-including HCMV- in recipient cells by stimulating autophagy [70]. Pegtel *et al*. [71] recently demonstrated that EBV miRNAs produced by EBV-infected cells are transferred via exosomes to uninfected cells where they are able to down-regulate known targets. Interestingly, significantly higher level of miR-UL112-3p has been detected in the serum of patients with essential hypertension compared to healthy patients [72], a finding that correlated with elevated serum level of HCMV DNA. Combined with the fact that seropositivity rate was similar between the hypertensive and healthy group of patients, these data suggest that miR-UL112-3p is secreted from cells supporting active viral replication into the plasma. This opens the possibility that miR-UL112-3p produced in infected cells might target TLR2 expression in neighboring non-infected cells. Such paracrine down-regulation of TLR2 could curtail the initial steps of innate signaling and antiviral defense triggered by HCMV gB and gH binding to TLR2, facilitating HCMV infection of neighboring cells and blunting activation of NK cells. Additional studies will be required to investigate these exciting possibilities.

## Materials and Methods

### Cells and media

HEK293T (ATCC #CRL-11268) and adult normal human fibroblasts (NHDF) (Lonza #CC-2511) were cultured in DMEM. THP-1 (ATCC #TIB-202) cells were cultured in RPMI. All cells were cultured at 37°C in 5% CO2 in media containing 10% fetal bovine serum and penicillin/streptomycin. Stable NHDF-NFκBRE-luc and THP-1-NFκBRE-luc cells were cultured in media supplemented with 3ug/ml puromycin.

### Plasmids, siRNAs, miRNAs, chemicals

The 3'UTR of human CD14, TLR1, TLR2, TLR3, TLR9, Rac1, Rac2, Rac3, TRIF/TICAM1 and TIRP/TICAM2 were amplified by RT-PCR from total RNA preps extracted from HEK293 cells, TPA-differentiated THP1 cells or primary monocytes using Trizol (Life Technologies). XhoI and NotI restriction sites were added at the 5' end of the forward and reverse PCR primers, respectively, allowing to clone PCR products downstream of the Renilla luciferase gene in the pSICHECK2 plasmid (Promega) using the same restriction sites. Mutations in the seed sequence of putative miR-UL112-3p target sites were introduced in the pSICHECK2-TLR2 3'UTR construct by site-directed mutagenesis using the following primers pairs (mutated nucleotides are bolded):

For miR-UL112-3p target site #1:

CCAGTCTTTGTCTAGTTGGGATCTTTATG**CA**A**G**TAGTTATAGTTAAGTTCATTCAGAC, and GTCTGAATGAACTTAACTATAACTA**C**T**TG**CATAAAGATCCCAACTAGACAAAGACTGG.

For miR-UL112-3p target site #2:

GGATGTACCG**GT**ATTTGAGGACTTGCTTACTAAAAC, and GTCCTCAAAT**AC**CGGTACATCCACGTAGTTTTTAT.

All mutated constructs were verified by sequencing.

### Reagents and antibodies

Silencer Select Negative control #1 (NEG, #4390843) and TLR2 siRNA (#s168) were from Ambion/Life Technologies; double stranded miRNA mimics of miR-UL112-3p, miR-US25-1 and miR-US25-2 were designed using the published miRNA sequences (www.mirbase.org) as described previously [25,28] and custom synthetized by IDT (Integrated DNA Technologies).

TLR2 agonists PAM3CSK4 and FSL-1, and TLR4 agonist LPS (all from Invivogen) were dissolved in water. The following commercial antibodies were used: β-actin (#MAB1501R, Millipore), GAPDH (#ab8245, Abcam), IRAK1 (#PA5-17490, Pierce), TIRP/TICAM2 (#TA306163, Origene), TLR2 (#3268–1, Epitomics), MyD88 (#ab2064, Abcam) and TRAM1 (#TA308042, Origene). Monoclonal pp65 (28–19) and pp28 (41–18) antibodies were a kind gift from W. Britt (U. of Alabama Birmingham). Rabbit polyclonal HCMV IE2 antibody (R638) was raised using bacterially-expressed whole IE2 protein and recognizes several IE products including IE1 and IE2 [23].

### HCMV constructs

AD169 112KO and TKO (miR-US5-1, miR-US5-2, miR-UL112-3p KO) mutants have been described in [32]. HCMV TB40E has been described in [73]. UV inactivation was performed as described [74].

### pSICHECK2 and NFκBRE reporter luciferase assays

pSICHECK2 transfections were performed in 96-well format. In each well, 1x10^4^ HEK293 cells were co-transfected with 50ng pSICHECK2 plasmid and 0.3pmol miRNA mimic (or 0.05pmol when indicated) using 0.3ul lipofectamine 2000 (Life Technologies). Transfection of THP-1- NFκBRE-luc cells [differentiated for 24hrs with 100ng/ml 12-*O*-Tetradecanoylphorbol-13-acetate (TPA)] was performed in 96-well format (2x10^4^ cells/well) using 3pmol miRNA or siRNA and 0.25ul lipofectamine RNAiMAX (Life Technologies). Differentiated THP1 or NHDF cells were stimulated with TLR agonists as indicated in figure legends. Luciferase assays were performed as described [25].

### TLR2 down-regulation and IRAK1 activation assays

NHDF cells plated in 48-well culture plates were infected with virus/MOI indicated in figure legends for 4 hours before inoculum was replaced with fresh media. For timecourse experiments, cells were harvested at various times post-infection and analyzed by IB and miRNA RT-PCR. THP-1 cells were differentiated with TPA 24hrs prior to transfections. For siRNA/miRNA transfection analysis, each well was transfected with 0.5ul lipofectamine RNAiMax and 2pmol siRNA or miRNA. In infection/transfection experiments, transfections were performed 4 hrs post-infection. When indicated, cells were stimulated with TLR agonists (FSL1, PAM3CSK4, LPS) or water before IB analysis.

### Immunoblots

Cells were harvested in protein lysis buffer (50mM Tris-HCl pH 6.8, 2% SDS, 20% glycerol). Cell lysates containing 20ug total proteins were incubated at 95°C for 10 min, loaded onto 9% Tris-Glycine SDS-PAGE gel along with prestained protein markers, and electrophoresed for 45min at 150V. After wet transfer (1hr, 100V), PVDF membranes were blocked in TBST / 5% nonfat milk then probed with primary and secondary HRP antibodies following manufacturer/'s recommendations. Protein signals were revealed using the ECL plus kit (Pierce) and exposure to chemiluminescent photographic films. Bands were quantified using the ImageJ software [75].

### RT-qPCR

#### miR-UL112-3p

Total RNA was harvested using Trizol and reverse transcribed using a miR-UL112-3p-specific stem-loop RT primer: 5'GTCGTATCCAGTGCAGGGTCCGAGGTATTCGCACTGGATACGACAGCCTG 3'.

Primers (Fwd: CGCGCAAGTGACGGTGAGAT; Rev: GTGCAGGGTCCGAGGT) and a Taqman probe (6FAM)ATACGACAGCCTGGAT(MGB) (Taqman, Life Sciences) were then used for real-time PCR amplification. miR-UL112-3p copy numbers in samples were determined from a standard curve established with serial dilutions of the miR-UL112-3p mimic.

#### GAPDH, TLR2, IL-1β, IL-6, IL-8

Total RNAs were reverse transcribed using a mix of random hexanucleotide primers and qPCR was performed using the following commercial Taqman assays (Life Technologies): Hs02758991_g1 (GAPDH), Hs00985639_m1 (IL-6), Hs00174103_m1 (IL-8), Hs01555410_m1 (IL-1β), Hs00610101_m1 (TLR2). For each sample, ΔΔCT relative quantitation of TLR2, IL-1β, IL-6 and IL-8 was performed using GAPDH as reference.

### List of genes cited in the manuscript

Human TLR2, UniProt O60603
Human IRAK1, UniProt P51617
Human IL-1β, UniProt P01584
Human IL-6, UniProt P05231
Human IL-8, UniProt P10145
Human GAPDH, UniProt P04406
Human TRAM1, UniProt Q15629
Human CD14, UniProt P08571
Human TLR1, UniProt Q15399
Human TLR3, UniProt O15455
HumanTLR9, UniProt Q9NR96
Human Rac1, UniProt P63000
Human Rac2, UniProt P15153
Human Rac3, UniProt P60763
Human TRIF/TICAM1, UniProt Q8IUC6
Human TIRP/TICAM2, UniProt Q86XR7
Human β-actin, UniProt P60709
HCMV IE1, UniProt P03169
HCMV IE2, UniProt P19893
HCMV pp28, UniProt P13200
HCMV pp65, UniProt P06725
HCMV miR-UL22A-5p, MirBase MIMAT0001574
HCMV miR-US25-2-5p, MirBase MIMAT0001582
HCMV miR-US25-2-3p, MirBase MIMAT0001582
HCMV miR-UL112-3p, MirBase MIMAT0001577
HCMV miR-US5-1, MirBase MIMAT0001579
HCMV miR-US5-2-3p, MirBase MIMAT0001580
HCMV miR-US25-1-5p, MirBase MIMAT0001581
HCMV miR-US33-5p, MirBase MIMAT0001584
HCMV miR-UL36-5p, MirBase MIMAT0001576
HCMV miR-UL148D, MirBase MIMAT0001578

## Supporting Information

S1 Fig Reactome pathway enrichment analysis and luciferase assay screen to identify TLR pathway genes targeted by HCMV miRNAs. Upper panel: Analysis of the Reactome database (www.reactome.org) for pathways enriched in HCMV miRNA target sites identified TLR signaling-related cellular pathways. Briefly, for each pathway in the reactome database we generated 1,000 mock pathways of the same gene size and identified the presence of HCMV miRNA target sites in the 3'UTR of each gene member of these pathways (real and mock). For each miRNA, we then determined the distribution [number of pathways vs. number of targeted genes] among the 1,000 mock pathways. Reactome pathways that were in the 99 percentile of the distribution (i.e. that contained a number of miRNA target sites that fit them in the top 1% of pathway most enriched in miRNA target sites) were considered potential targets of HCMV miRNAs. Middle panel: flow diagram of the dual luciferase reporter assay. Lower panels: Luciferase assays were performed on two independent pSICHECK2 3’UTR clones for each TLR pathway gene tested (blue and purple bars), except Rac1 and Rac2 (one clone tested) using mimics of 10 confirmed HCMV miRNAs (miR-US5-1, miR-US5-2, miR-UL22A, miR-US25-1, miR-US25-2 5p, miR-US25-2 3p, miR-US33, miR-UL36, miR-UL112-3p, miR-UL148D) and 2 putative miRNAs (miR-UL63 and miR-UL148D*). NEG, non-targeting miRNA mimic. Blue arrows indicate possible miRNA mimic downregulation of the reporter construct. Putative and confirmed HCMV miRNAs are indicated in abbreviated form.
(TIF)

S2 Fig Confirmatory screens for selected TLR genes and miRNAs. Luciferase assays were performed with the indicated pSICHECK2 constructs using two miRNA doses, alone or in combination as indicated.
(TIF)

S3 Fig A miR-UL112-3p mimic down-regulates endogenous TLR2 in NHDF fibroblast cells as efficiently as a commercial TLR2 siRNA. NHDF cells infected with AD169 (MOI: 1) and transfected 4 hrs later with various siRNA and miRNA mimics were harvested 2 days post-infection for IB analysis. Numbers below the TLR2 and MyD88 blots represent quantification (in relative units) of the protein signal.
(TIF)

S4 Fig TLR2 upregulation during HCMV infection of fibroblasts requires live virus. NHDF cells were infected with UV-inactivated or live AD169 WT, miR-UL112-3p KO at MOI: 1. Cells were harvested at 2 dpi and analyzed by IB. *, non-specific product.
(TIF)

S5 Fig TLR2 accumulates during infection with an AD169 triple mutant virus (TKO, miR-US5-1, miR-US5-2, miR-UL112-3p KO). NHDF cells were infected with AD169 WT or AD169 TKO at MOI: 3 and harvested at indicated times for IB analysis. Numbers below the TLR2 blot represent quantification (in relative units) of the protein signal. Detection of the tegument protein pp28 at 8hpi in AD169 TKO-infected cells may be due to an abundance of pp28-containing noninfectious particles in the inoculum: the TKO virus has a marked growth defect compared to the WT virus, causing the generation of a higher proportion of noninfectious particles [32] that contain the tegument protein pp28. The quantity of AD169 TKO inoculum necessary to reach a MOI of 3 may therefore contain more defective particles than the WT virus and hence more pp28. Note that pp65, another tegument protein, does not show the same pattern. This could either be due to a lack of sensitivity of the antibody or differential enrichment of pp28 and pp65 in AD169 TKO noninfectious particles.
(TIF)

S6 Fig THP1 but not NHDF cells are responsive to stimulation of the NFκB pathway by the TLR2 agonist PAM3CSK4. NFκBRE luciferase reporter cells (NHDF fibroblasts or THP-1 cells differentiated with TPA) were stimulated with PAM3CSK4 (TLR2/TLR1 agonist, 100 ng/ml) or LPS (TLR4 agonist, 1 ug/ml) for 6 hrs and NFκB activation measured by luciferase assay.
(TIF)

S7 Fig miR-UL112-3p does not target the unmodified form of IRAK1. THP1 cells were transfected for 2 days with 33nM of the indicated siRNA or miRNAs before harvest and IB analysis.
(TIF)

## References

[1] Akira S, Uematsu S, Takeuchi O. Pathogen recognition and innate immunity. Cell. 2006;124: 783–801. 16497588 10.1016/j.cell.2006.02.015

[2] Kawai T, Akira S. The role of pattern-recognition receptors in innate immunity: update on Toll-like receptors. Nat Immunol. 2010;11: 373–384. 10.1038/ni.1863 20404851

[3] Oliveira-Nascimento L, Massari P, Wetzler LM. The Role of TLR2 in Infection and Immunity. Front Immunol. 2012;3: 79 10.3389/fimmu.2012.00079 22566960 PMC3342043

[4] Ariza ME, Glaser R, Kaumaya PT, Jones C, Williams MV. The EBV-encoded dUTPase activates NF-kappa B through the TLR2 and MyD88-dependent signaling pathway. J Immunol. 2009;182: 851–859. 19124728 10.4049/jimmunol.182.2.851 PMC12892303

[5] Chang S, Dolganiuc A, Szabo G. Toll-like receptors 1 and 6 are involved in TLR2-mediated macrophage activation by hepatitis C virus core and NS3 proteins. J Leukoc Biol. 2007;82: 479–487. 17595379 10.1189/jlb.0207128

[6] Compton T, Kurt-Jones EA, Boehme KW, Belko J, Latz E, Golenbock DT, et al Human cytomegalovirus activates inflammatory cytokine responses via CD14 and Toll-like receptor 2. J Virol. 2003;77: 4588–4596. 12663765 10.1128/JVI.77.8.4588-4596.2003 PMC152130

[7] Boehme KW, Guerrero M, Compton T. Human cytomegalovirus envelope glycoproteins B and H are necessary for TLR2 activation in permissive cells. J Immunol. 2006;177: 7094–7102. 17082626 10.4049/jimmunol.177.10.7094

[8] Barbalat R, Lau L, Locksley RM, Barton GM. Toll-like receptor 2 on inflammatory monocytes induces type I interferon in response to viral but not bacterial ligands. Nat Immunol. 2009;10: 1200–1207. 10.1038/ni.1792 19801985 PMC2821672

[9] Kijpittayarit S, Eid AJ, Brown RA, Paya CV, Razonable RR. Relationship between Toll-like receptor 2 polymorphism and cytomegalovirus disease after liver transplantation. Clin Infect Dis. 2007;44: 1315–1320. 17443468 10.1086/514339

[10] von Aulock S, Schroder NW, Traub S, Gueinzius K, Lorenz E, Hartung T, et al Heterozygous toll-like receptor 2 polymorphism does not affect lipoteichoic acid-induced chemokine and inflammatory responses. Infect Immun. 2004;72: 1828–1831. 14977997 10.1128/IAI.72.3.1828-1831.2004 PMC356018

[11] Hamann L, Gomma A, Schroder NW, Stamme C, Glaeser C, Schulz S, et al A frequent toll-like receptor (TLR)-2 polymorphism is a risk factor for coronary restenosis. J Mol Med (Berl). 2005;83: 478–485. 15875151 10.1007/s00109-005-0643-7

[12] Kang SH, Abdel-Massih RC, Brown RA, Dierkhising RA, Kremers WK, Razonable RR. Homozygosity for the toll-like receptor 2 R753Q single-nucleotide polymorphism is a risk factor for cytomegalovirus disease after liver transplantation. J Infect Dis. 2012;205: 639–646. 10.1093/infdis/jir819 22219347 PMC3266129

[13] Xiong Y, Song C, Snyder GA, Sundberg EJ, Medvedev AE. R753Q polymorphism inhibits Toll-like receptor (TLR) 2 tyrosine phosphorylation, dimerization with TLR6, and recruitment of myeloid differentiation primary response protein 88. J Biol Chem. 2012;287: 38327–38337. 10.1074/jbc.M112.375493 22992740 PMC3488101

[14] Szomolanyi-Tsuda E, Liang X, Welsh RM, Kurt-Jones EA, Finberg RW. Role for TLR2 in NK cell-mediated control of murine cytomegalovirus in vivo. J Virol. 2006;80: 4286–4291. 16611887 10.1128/JVI.80.9.4286-4291.2006 PMC1472014

[15] Bartel DP. MicroRNAs: target recognition and regulatory functions. Cell. 2009;136: 215–233. 10.1016/j.cell.2009.01.002 19167326 PMC3794896

[16] Pfeffer S, Zavolan M, Grasser FA, Chien M, Russo JJ, Ju J, et al Identification of virus-encoded microRNAs. Science. 2004;304: 734–736. 15118162 10.1126/science.1096781

[17] Grey F, Antoniewicz A, Allen E, Saugstad J, McShea A, Carrington JC, et al Identification and characterization of human cytomegalovirus-encoded microRNAs. J Virol. 2005;79: 12095–12099. 16140786 10.1128/JVI.79.18.12095-12099.2005 PMC1212634

[18] Stark TJ, Arnold JD, Spector DH, Yeo GW. High-resolution profiling and analysis of viral and host small RNAs during human cytomegalovirus infection. J Virol. 2012;86: 226–235. 10.1128/JVI.05903-11 22013051 PMC3255895

[19] Grey F. The role of microRNAs in herpesvirus latency and persistence. J Gen Virol. 2014. 10.1099/vir.0.070862-0 25406174

[20] Hook L, Hancock M, Landais I, Grabski R, Britt W, Nelson JA. Cytomegalovirus microRNAs. Curr Opin Virol. 2014;7: 40–46. 10.1016/j.coviro.2014.03.015 24769092 PMC4149926

[21] Nachmani D, Stern-Ginossar N, Sarid R, Mandelboim O. Diverse herpesvirus microRNAs target the stress-induced immune ligand MICB to escape recognition by natural killer cells. Cell Host Microbe. 2009;5: 376–385. 10.1016/j.chom.2009.03.003 19380116

[22] Gottwein E, Corcoran DL, Mukherjee N, Skalsky RL, Hafner M, Nusbaum JD, et al Viral microRNA targetome of KSHV-infected primary effusion lymphoma cell lines. Cell Host Microbe. 2011;10: 515–526. 10.1016/j.chom.2011.09.012 22100165 PMC3222872

[23] Grey F, Meyers H, White EA, Spector DH, Nelson J. A human cytomegalovirus-encoded microRNA regulates expression of multiple viral genes involved in replication. PLoS Pathog. 2007;3: e163 17983268 10.1371/journal.ppat.0030163 PMC2048532

[24] Umbach JL, Kramer MF, Jurak I, Karnowski HW, Coen DM, Cullen BR. MicroRNAs expressed by herpes simplex virus 1 during latent infection regulate viral mRNAs. Nature. 2008;454: 780–783. 10.1038/nature07103 18596690 PMC2666538

[25] Tirabassi R, Hook L, Landais I, Grey F, Meyers H, Hewitt H, et al Human cytomegalovirus US7 is regulated synergistically by two virally encoded microRNAs and by two distinct mechanisms. J Virol. 2011;85: 11938–11944. 10.1128/JVI.05443-11 21900172 PMC3209316

[26] Samols MA, Skalsky RL, Maldonado AM, Riva A, Lopez MC, Baker HV, et al Identification of cellular genes targeted by KSHV-encoded microRNAs. PLoS Pathog. 2007;3: e65 17500590 10.1371/journal.ppat.0030065 PMC1876501

[27] Ziegelbauer JM, Sullivan CS, Ganem D. Tandem array-based expression screens identify host mRNA targets of virus-encoded microRNAs. Nat Genet. 2009;41: 130–134. 10.1038/ng.266 19098914 PMC2749995

[28] Grey F, Tirabassi R, Meyers H, Wu G, McWeeney S, Hook L, et al A viral microRNA down-regulates multiple cell cycle genes through mRNA 5'UTRs. PLoS Pathog. 2010;6: e1000967 10.1371/journal.ppat.1000967 20585629 PMC2891821

[29] Lei X, Bai Z, Ye F, Xie J, Kim CG, Huang Y, et al Regulation of NF-kappaB inhibitor IkappaBalpha and viral replication by a KSHV microRNA. Nat Cell Biol. 2010;12: 193–199. 10.1038/ncb2019 20081837 PMC2815189

[30] Lee SH, Kalejta RF, Kerry J, Semmes OJ, O'Connor CM, Khan Z, et al BclAF1 restriction factor is neutralized by proteasomal degradation and microRNA repression during human cytomegalovirus infection. Proc Natl Acad Sci U S A. 2012;109: 9575–9580. 10.1073/pnas.1207496109 22645331 PMC3386064

[31] Skalsky RL, Corcoran DL, Gottwein E, Frank CL, Kang D, Hafner M, et al The viral and cellular microRNA targetome in lymphoblastoid cell lines. PLoS Pathog. 2012;8: e1002484 10.1371/journal.ppat.1002484 22291592 PMC3266933

[32] Hook LM, Grey F, Grabski R, Tirabassi R, Doyle T, Hancock M, et al Cytomegalovirus miRNAs target secretory pathway genes to facilitate formation of the virion assembly compartment and reduce cytokine secretion. Cell Host Microbe. 2014;15: 363–373. 10.1016/j.chom.2014.02.004 24629342 PMC4029511

[33] Kim Y, Lee S, Kim S, Kim D, Ahn JH, Ahn K. Human cytomegalovirus clinical strain-specific microRNA miR-UL148D targets the human chemokine RANTES during infection. PLoS Pathog. 2012;8: e1002577 10.1371/journal.ppat.1002577 22412377 PMC3297591

[34] Stern-Ginossar N, Elefant N, Zimmermann A, Wolf DG, Saleh N, Biton M, et al Host immune system gene targeting by a viral miRNA. Science. 2007;317: 376–381. 17641203 10.1126/science.1140956 PMC4283197

[35] Nachmani D, Lankry D, Wolf DG, Mandelboim O. The human cytomegalovirus microRNA miR-UL112 acts synergistically with a cellular microRNA to escape immune elimination. Nat Immunol. 2010;11: 806–813. 10.1038/ni.1916 20694010

[36] Abend JR, Ramalingam D, Kieffer-Kwon P, Uldrick TS, Yarchoan R, Ziegelbauer JM. Kaposi's sarcoma-associated herpesvirus microRNAs target IRAK1 and MYD88, two components of the toll-like receptor/interleukin-1R signaling cascade, to reduce inflammatory-cytokine expression. J Virol. 2012;86: 11663–11674. 10.1128/JVI.01147-12 22896623 PMC3486292

[37] Chen Y, Chen J, Wang H, Shi J, Wu K, Liu S, et al HCV-induced miR-21 contributes to evasion of host immune system by targeting MyD88 and IRAK1. PLoS Pathog. 2013;9: e1003248 10.1371/journal.ppat.1003248 23633945 PMC3635988

[38] Benakanakere MR, Li Q, Eskan MA, Singh AV, Zhao J, Galicia JC, et al Modulation of TLR2 protein expression by miR-105 in human oral keratinocytes. J Biol Chem. 2009;284: 23107–23115. 10.1074/jbc.M109.013862 19509287 PMC2755716

[39] Philippe L, Alsaleh G, Suffert G, Meyer A, Georgel P, Sibilia J, et al TLR2 expression is regulated by microRNA miR-19 in rheumatoid fibroblast-like synoviocytes. J Immunol. 2012;188: 454–461. 10.4049/jimmunol.1102348 22105995

[40] Guo H, Chen Y, Hu X, Qian G, Ge S, Zhang J. The regulation of Toll-like receptor 2 by miR-143 suppresses the invasion and migration of a subset of human colorectal carcinoma cells. Mol Cancer. 2013;12: 77 10.1186/1476-4598-12-77 23866094 PMC3750391

[41] Sallustio F, Serino G, Costantino V, Curci C, Cox SN, De Palma G, et al miR-1915 and miR-1225-5p regulate the expression of CD133, PAX2 and TLR2 in adult renal progenitor cells. PLoS One. 2013;8: e68296 10.1371/journal.pone.0068296 23861881 PMC3704645

[42] Xin C, Zhang H, Liu Z. miR-154 suppresses colorectal cancer cell growth and motility by targeting TLR2. Mol Cell Biochem. 2014;387: 271–277. 10.1007/s11010-013-1892-3 24242044

[43] Croft D, Mundo AF, Haw R, Milacic M, Weiser J, Wu G, et al The Reactome pathway knowledgebase. Nucleic Acids Res. 2014;42: D472–477. 10.1093/nar/gkt1102 24243840 PMC3965010

[44] Browne EP, Wing B, Coleman D, Shenk T. Altered cellular mRNA levels in human cytomegalovirus-infected fibroblasts: viral block to the accumulation of antiviral mRNAs. J Virol. 2001;75: 12319–12330. 11711622 10.1128/JVI.75.24.12319-12330.2001 PMC116128

[45] Chan G, Guilbert LJ. Ultraviolet-inactivated human cytomegalovirus induces placental syncytiotrophoblast apoptosis in a Toll-like receptor-2 and tumour necrosis factor-alpha dependent manner. J Pathol. 2006;210: 111–120. 16826536 10.1002/path.2025

[46] Lee RM, White MR, Hartshorn KL. Influenza a viruses upregulate neutrophil toll-like receptor 2 expression and function. Scand J Immunol. 2006;63: 81–89. 16476006 10.1111/j.1365-3083.2005.01714.x

[47] Villalba M, Hott M, Martin C, Aguila B, Valdivia S, Quezada C, et al Herpes simplex virus type 1 induces simultaneous activation of Toll-like receptors 2 and 4 and expression of the endogenous ligand serum amyloid A in astrocytes. Med Microbiol Immunol. 2012;201: 371–379. 10.1007/s00430-012-0247-0 22622619

[48] Murphy E, Vanicek J, Robins H, Shenk T, Levine AJ. Suppression of immediate-early viral gene expression by herpesvirus-coded microRNAs: implications for latency. Proc Natl Acad Sci U S A. 2008;105: 5453–5458. 10.1073/pnas.0711910105 18378902 PMC2291141

[49] Jarvis MA, Borton JA, Keech AM, Wong J, Britt WJ, Magun BE, et al Human cytomegalovirus attenuates interleukin-1beta and tumor necrosis factor alpha proinflammatory signaling by inhibition of NF-kappaB activation. J Virol. 2006;80: 5588–5598. 16699040 10.1128/JVI.00060-06 PMC1472148

[50] Yew KH, Carpenter C, Duncan RS, Harrison CJ. Human cytomegalovirus induces TLR4 signaling components in monocytes altering TIRAP, TRAM and downstream interferon-beta and TNF-alpha expression. PLoS One. 2012;7: e44500 10.1371/journal.pone.0044500 22970235 PMC3436894

[51] Harwani SC, Lurain NS, Zariffard MR, Spear GT. Differential inhibition of human cytomegalovirus (HCMV) by toll-like receptor ligands mediated by interferon-beta in human foreskin fibroblasts and cervical tissue. Virol J. 2007;4: 133 18053251 10.1186/1743-422X-4-133 PMC2222636

[52] Wang JP, Kurt-Jones EA, Shin OS, Manchak MD, Levin MJ, Finberg RW. Varicella-zoster virus activates inflammatory cytokines in human monocytes and macrophages via Toll-like receptor 2. J Virol. 2005;79: 12658–12666. 16188968 10.1128/JVI.79.20.12658-12666.2005 PMC1235827

[53] Gutzeit C, Raftery MJ, Peiser M, Tischer KB, Ulrich M, Eberhardt M, et al Identification of an important immunological difference between virulent varicella-zoster virus and its avirulent vaccine: viral disruption of dendritic cell instruction. J Immunol. 2010;185: 488–497. 10.4049/jimmunol.0902817 20525895 PMC3033232

[54] Sen J, Liu X, Roller R, Knipe DM. Herpes simplex virus US3 tegument protein inhibits Toll-like receptor 2 signaling at or before TRAF6 ubiquitination. Virology. 2013;439: 65–73. 10.1016/j.virol.2013.01.026 23478027 PMC3810314

[55] Durantel D, Zoulim F. Interplay between hepatitis B virus and TLR2-mediated innate immune responses: can restoration of TLR2 functions be a new therapeutic option? J Hepatol. 2012;57: 486–489. 10.1016/j.jhep.2012.06.019 22728561

[56] Wang S, Chen Z, Hu C, Qian F, Cheng Y, Wu M, et al Hepatitis B virus surface antigen selectively inhibits TLR2 ligand-induced IL-12 production in monocytes/macrophages by interfering with JNK activation. J Immunol. 2013;190: 5142–5151. 10.4049/jimmunol.1201625 23585678

[57] Yao XD, Rosenthal KL. Herpes simplex virus type 2 virion host shutoff protein suppresses innate dsRNA antiviral pathways in human vaginal epithelial cells. J Gen Virol. 2011;92: 1981–1993. 10.1099/vir.0.030296-0 21632561

[58] Saetrom P, Heale BS, Snove O Jr., Aagaard L, Alluin J, Rossi JJ. Distance constraints between microRNA target sites dictate efficacy and cooperativity. Nucleic Acids Res. 2007;35: 2333–2342. 17389647 10.1093/nar/gkm133 PMC1874663

[59] Brown RA, Gralewski JH, Razonable RR. The R753Q polymorphism abrogates toll-like receptor 2 signaling in response to human cytomegalovirus. Clin Infect Dis. 2009;49: e96–99. 10.1086/644501 19814623 PMC2761976

[60] Leoni V, Gianni T, Salvioli S, Campadelli-Fiume G. Herpes simplex virus glycoproteins gH/gL and gB bind Toll-like receptor 2, and soluble gH/gL is sufficient to activate NF-kappaB. J Virol. 2012;86: 6555–6562. 10.1128/JVI.00295-12 22496225 PMC3393584

[61] Aliprantis AO, Yang RB, Weiss DS, Godowski P, Zychlinsky A. The apoptotic signaling pathway activated by Toll-like receptor-2. EMBO J. 2000;19: 3325–3336. 10880445 10.1093/emboj/19.13.3325 PMC313930

[62] Salaun B, Romero P, Lebecque S. Toll-like receptors' two-edged sword: when immunity meets apoptosis. Eur J Immunol. 2007;37: 3311–3318. 18034428 10.1002/eji.200737744

[63] Aravalli RN, Hu S, Lokensgard JR. Toll-like receptor 2 signaling is a mediator of apoptosis in herpes simplex virus-infected microglia. J Neuroinflammation. 2007;4: 11 17470292 10.1186/1742-2094-4-11 PMC1866225

[64] Chan G, Hemmings DG, Yurochko AD, Guilbert LJ. Human cytomegalovirus-caused damage to placental trophoblasts mediated by immediate-early gene-induced tumor necrosis factor-alpha. Am J Pathol. 2002;161: 1371–1381. 12368210 10.1016/s0002-9440(10)64413-6 PMC1867293

[65] Chaudhuri S, Lowen B, Chan G, Davey A, Riddell M, Guilbert LJ. Human cytomegalovirus interacts with toll-like receptor 2 and CD14 on syncytiotrophoblasts to stimulate expression of TNFalpha mRNA and apoptosis. Placenta. 2009;30: 994–1001. 10.1016/j.placenta.2009.09.001 19796811

[66] Goldmacher VS, Bartle LM, Skaletskaya A, Dionne CA, Kedersha NL, Vater CA, et al A cytomegalovirus-encoded mitochondria-localized inhibitor of apoptosis structurally unrelated to Bcl-2. Proc Natl Acad Sci U S A. 1999;96: 12536–12541. 10535957 10.1073/pnas.96.22.12536 PMC22976

[67] McCormick AL, Roback L, Livingston-Rosanoff D, St Clair C. The human cytomegalovirus UL36 gene controls caspase-dependent and-independent cell death programs activated by infection of monocytes differentiating to macrophages. J Virol. 2010;84: 5108–5123. 10.1128/JVI.01345-09 20219915 PMC2863825

[68] Pegtel DM, van de Garde MD, Middeldorp JM. Viral miRNAs exploiting the endosomal-exosomal pathway for intercellular cross-talk and immune evasion. Biochim Biophys Acta. 2011;1809: 715–721. 10.1016/j.bbagrm.2011.08.002 21855666

[69] van der Grein SG, Nolte-'t Hoen EN. "Small Talk" in the Innate Immune System via RNA-Containing Extracellular Vesicles. Front Immunol. 2014;5: 542 10.3389/fimmu.2014.00542 25400635 PMC4212677

[70] Delorme-Axford E, Donker RB, Mouillet JF, Chu T, Bayer A, Ouyang Y, et al Human placental trophoblasts confer viral resistance to recipient cells. Proc Natl Acad Sci U S A. 2013;110: 12048–12053. 10.1073/pnas.1304718110 23818581 PMC3718097

[71] Pegtel DM, Cosmopoulos K, Thorley-Lawson DA, van Eijndhoven MA, Hopmans ES, Lindenberg JL, et al Functional delivery of viral miRNAs via exosomes. Proc Natl Acad Sci U S A. 2010;107: 6328–6333. 10.1073/pnas.0914843107 20304794 PMC2851954

[72] Li S, Zhu J, Zhang W, Chen Y, Zhang K, Popescu LM, et al Signature microRNA expression profile of essential hypertension and its novel link to human cytomegalovirus infection. Circulation. 2011;124: 175–184. 10.1161/CIRCULATIONAHA.110.012237 21690488

[73] Sinzger C, Hahn G, Digel M, Katona R, Sampaio KL, Messerle M, et al Cloning and sequencing of a highly productive, endotheliotropic virus strain derived from human cytomegalovirus TB40/E. J Gen Virol. 2008;89: 359–368. 10.1099/vir.0.83286-0 18198366

[74] DeFilippis VR, Robinson B, Keck TM, Hansen SG, Nelson JA, Fruh KJ. Interferon regulatory factor 3 is necessary for induction of antiviral genes during human cytomegalovirus infection. J Virol. 2006;80: 1032–1037. 16379004 10.1128/JVI.80.2.1032-1037.2006 PMC1346858

[75] Schneider CA, Rasband WS, Eliceiri KW. NIH Image to ImageJ: 25 years of image analysis. Nat Methods. 2012;9: 671–675. 22930834 10.1038/nmeth.2089 PMC5554542

