# Supplementary figures and images for: Human Cytomegalovirus miR-UL112-3p Targets TLR2 and Modulates the TLR2/IRAK1/NFκB Signaling Pathway
Source: PLoS Pathog. 2015 May 8;11(5):e1004881. doi: 10.1371/journal.ppat.1004881 (PMC4425655; doi:10.1371/journal.ppat.1004881)

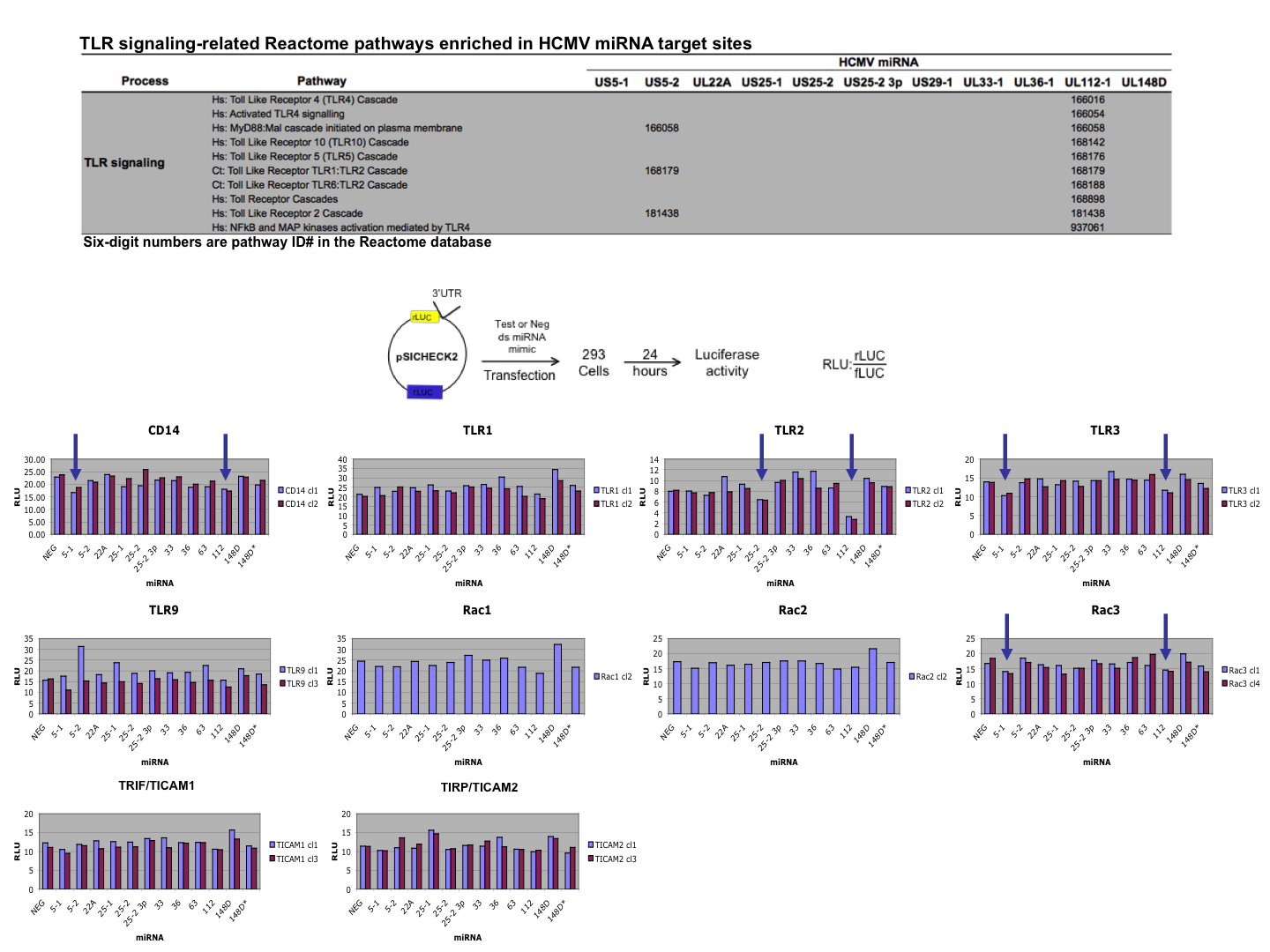

Supplement: S1 Fig — Upper panel: Analysis of the Reactome database (www.reactome.org) for pathways enriched in HCMV miRNA target sites identified TLR signaling-related cellular pathways. Briefly, for each pathway in the reactome database we generated 1,000 mock pathways of the same gene size and identified the presence of HCMV miRNA target sites in the 3'UTR of each gene member of these pathways (real and mock). For each miRNA, we then determined the distribution [number of pathways vs. number of targeted genes] among the 1,000 mock pathways. Reactome pathways that were in the 99 percentile of the distribution (i.e. that contained a number of miRNA target sites that fit them in the top 1% of pathway most enriched in miRNA target sites) were considered potential targets of HCMV miRNAs. Middle panel: flow diagram of the dual luciferase reporter assay. Lower panels: Luciferase assays were performed on two independent pSICHECK2 3’UTR clones for each TLR pathway gene tested (blue and purple bars), except Rac1 and Rac2 (one clone tested) using mimics of 10 confirmed HCMV miRNAs (miR-US5-1, miR-US5-2, miR-UL22A, miR-US25-1, miR-US25-2 5p, miR-US25-2 3p, miR-US33, miR-UL36, miR-UL112-3p, miR-UL148D) and 2 putative miRNAs (miR-UL63 and miR-UL148D*). NEG, non-targeting miRNA mimic. Blue arrows indicate possible miRNA mimic downregulation of the reporter construct. Putative and confirmed HCMV miRNAs are indicated in abbreviated form. (TIF) [file ppat.1004881.s001.tif]

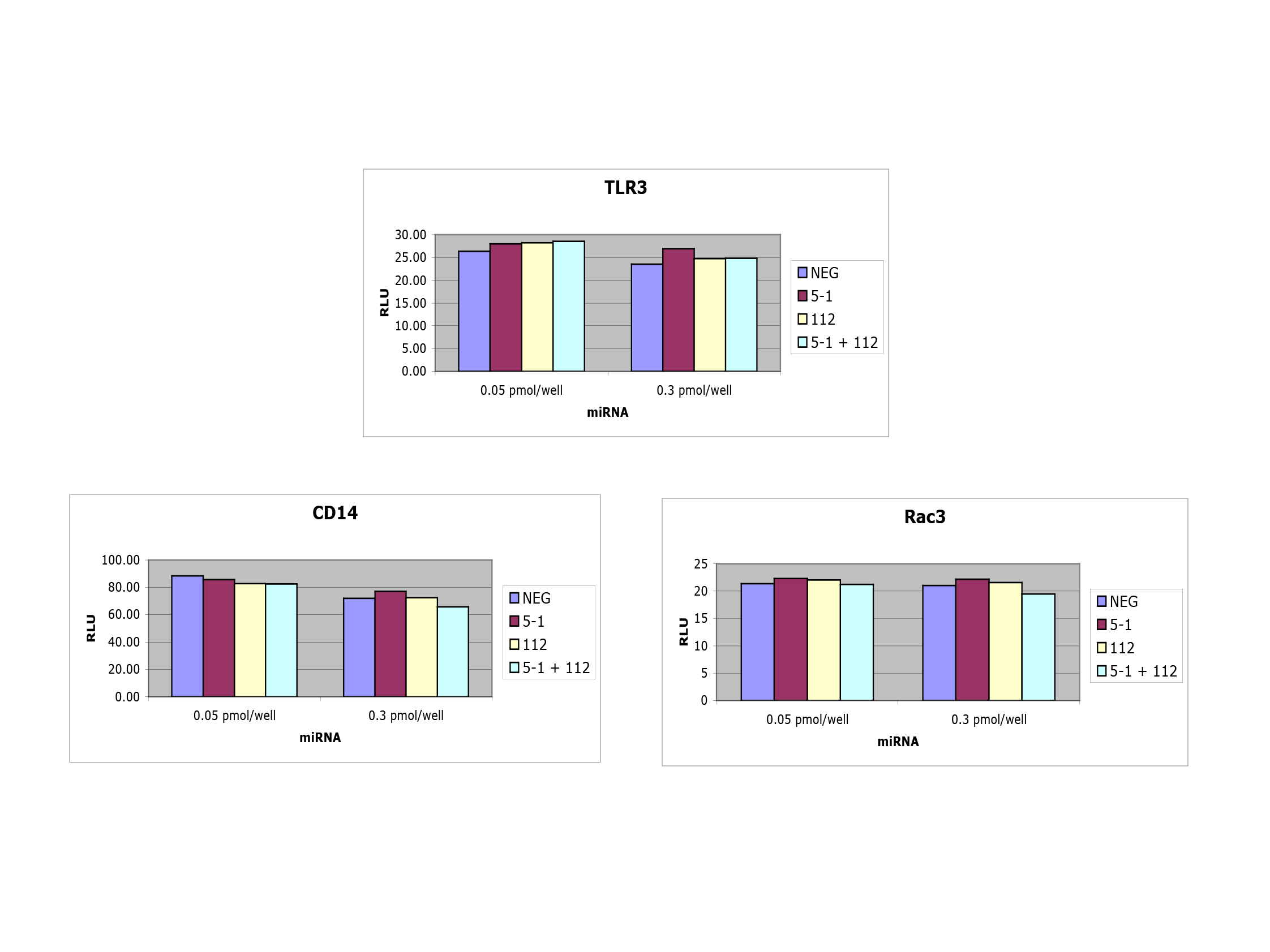

Supplement: S2 Fig — Luciferase assays were performed with the indicated pSICHECK2 constructs using two miRNA doses, alone or in combination as indicated. (TIF) [file ppat.1004881.s002.tif]

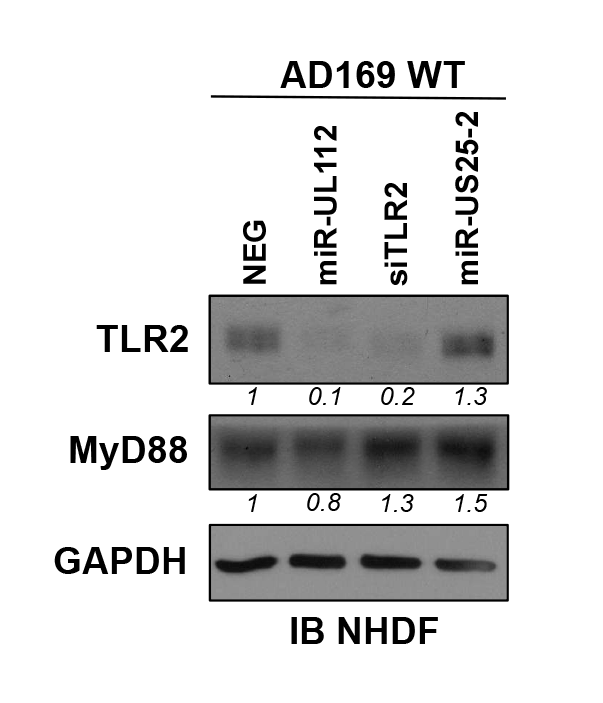

Supplement: S3 Fig — NHDF cells infected with AD169 (MOI: 1) and transfected 4 hrs later with various siRNA and miRNA mimics were harvested 2 days post-infection for IB analysis. Numbers below the TLR2 and MyD88 blots represent quantification (in relative units) of the protein signal. (TIF) [file ppat.1004881.s003.tif]

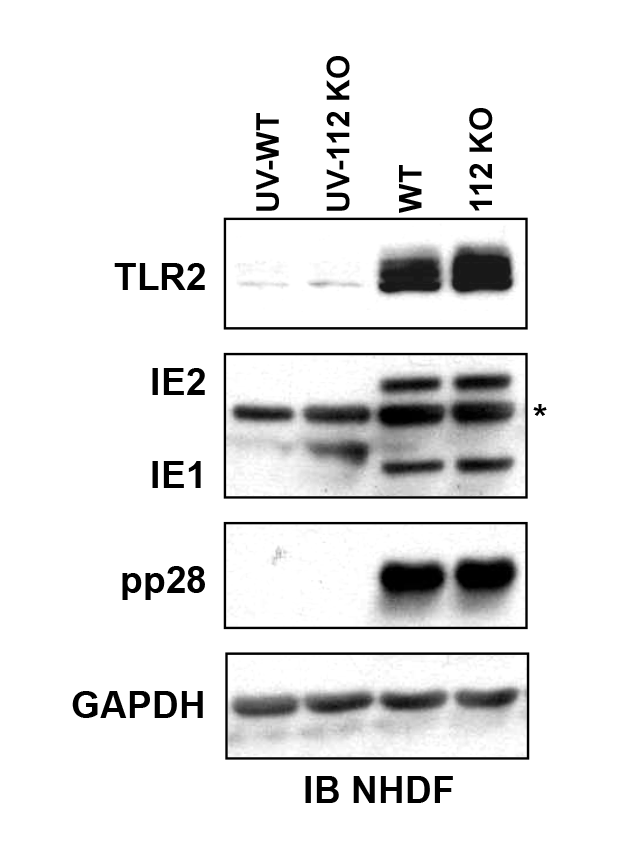

Supplement: S4 Fig — NHDF cells were infected with UV-inactivated or live AD169 WT, miR-UL112-3p KO at MOI: 1. Cells were harvested at 2 dpi and analyzed by IB. *, non-specific product. (TIF) [file ppat.1004881.s004.tif]

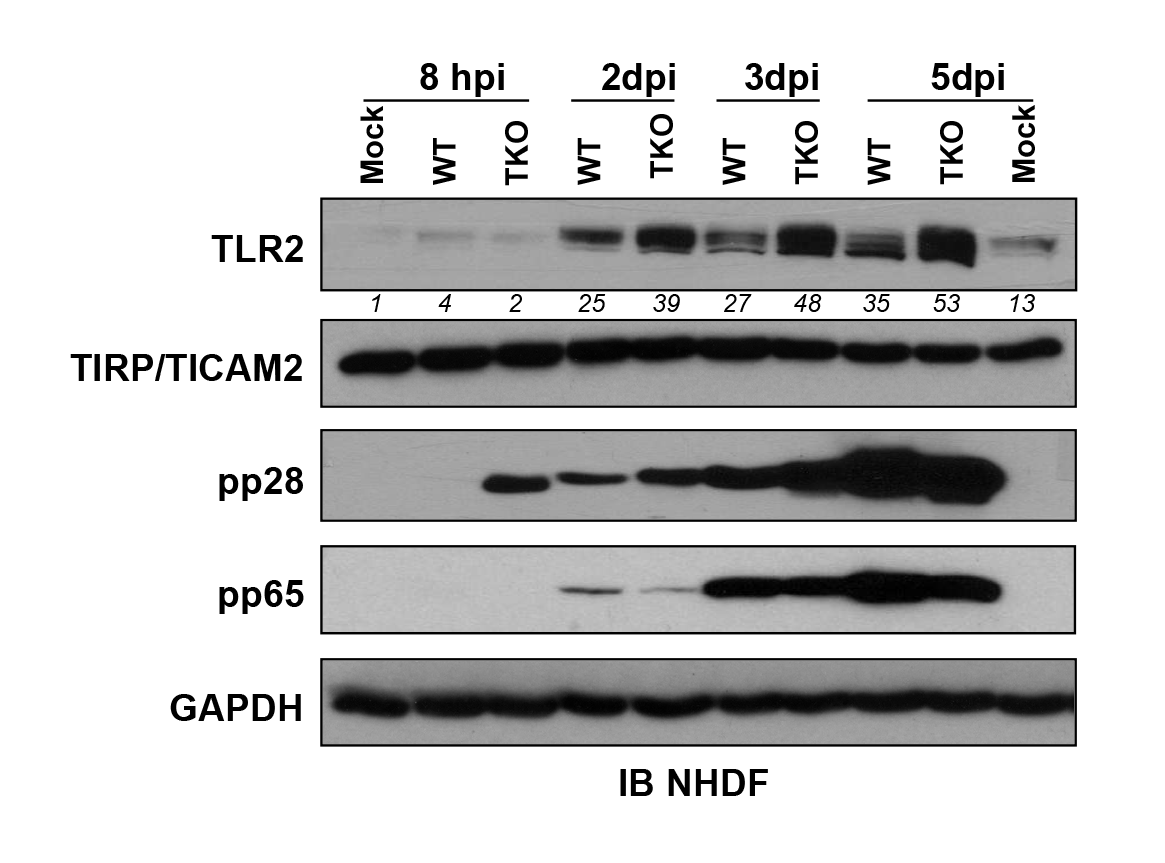

Supplement: S5 Fig — NHDF cells were infected with AD169 WT or AD169 TKO at MOI: 3 and harvested at indicated times for IB analysis. Numbers below the TLR2 blot represent quantification (in relative units) of the protein signal. Detection of the tegument protein pp28 at 8hpi in AD169 TKO-infected cells may be due to an abundance of pp28-containing noninfectious particles in the inoculum: the TKO virus has a marked growth defect compared to the WT virus, causing the generation of a higher proportion of noninfectious particles [32] that contain the tegument protein pp28. The quantity of AD169 TKO inoculum necessary to reach a MOI of 3 may therefore contain more defective particles than the WT virus and hence more pp28. Note that pp65, another tegument protein, does not show the same pattern. This could either be due to a lack of sensitivity of the antibody or differential enrichment of pp28 and pp65 in AD169 TKO noninfectious particles. (TIF) [file ppat.1004881.s005.tif]

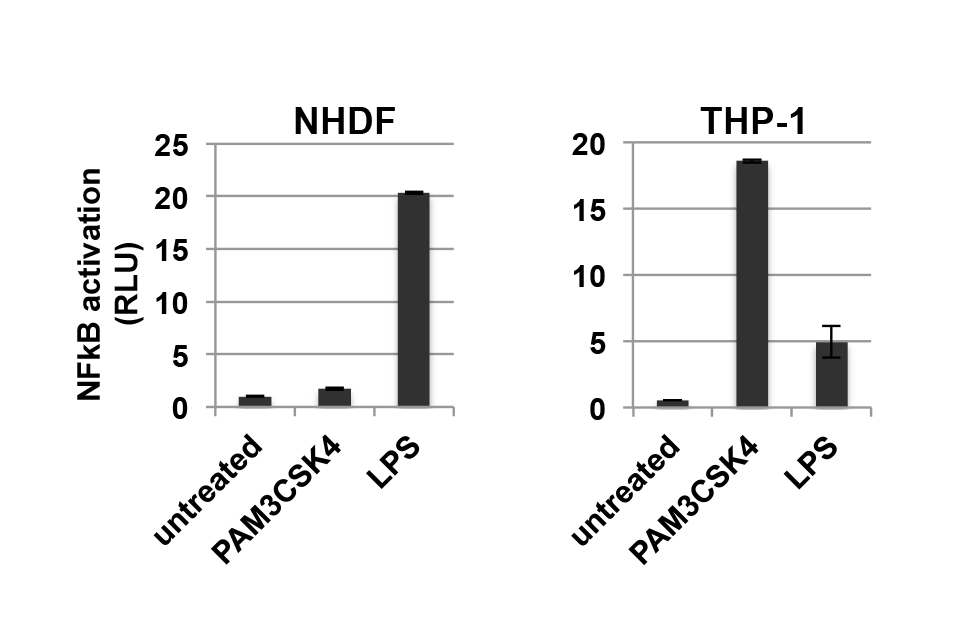

Supplement: S6 Fig — NFκBRE luciferase reporter cells (NHDF fibroblasts or THP-1 cells differentiated with TPA) were stimulated with PAM3CSK4 (TLR2/TLR1 agonist, 100 ng/ml) or LPS (TLR4 agonist, 1 ug/ml) for 6 hrs and NFκB activation measured by luciferase assay. (TIF) [file ppat.1004881.s006.tif]

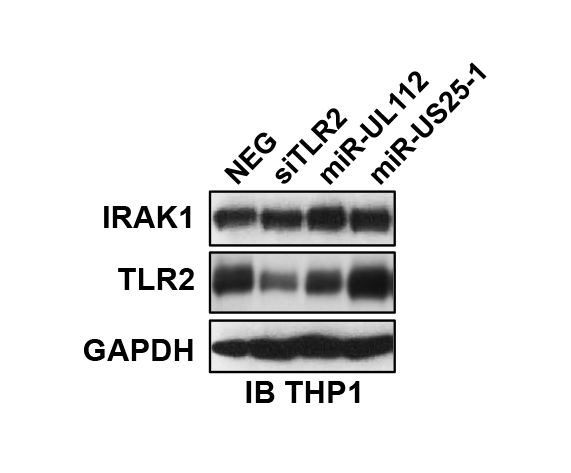

Supplement: S7 Fig — THP1 cells were transfected for 2 days with 33nM of the indicated siRNA or miRNAs before harvest and IB analysis. (TIF) [file ppat.1004881.s007.tif]
